# Outpatient compared to inpatient thyroidectomy on 30-day postoperative outcomes: a national propensity matched analysis

**DOI:** 10.1186/s13741-023-00335-x

**Published:** 2023-08-08

**Authors:** Lauren Traill, Mark C. Kendall, Maria Paula Caramez, Patricia Apruzzese, Gildasio De Oliveira

**Affiliations:** 1 Department of Anesthesiology, The Warren Alpert Medical School of Brown University, 593 Eddy Street, Providence, RI 02903 USA; 2 Department of Anesthesiology, Rhode Island Hospital, Providence, RI 02903 USA

**Keywords:** Thyroidectomy, Outpatient surgery, ACS NSQIP, Neck surgery, Postoperative complications

## Abstract

**Background:**

To address the postoperative outcomes between outpatient and inpatient neck surgery involving thyroidectomy procedures.

**Methods:**

A cohort analysis of surgical patients undergoing primary, elective, total thyroidectomy from multiple United States medical institutions who were registered with the American College of Surgeons National Surgical Quality Improvement Program from 2015 to 2018. The primary outcome was a composite score that included any 30-day postoperative adverse event.

**Results:**

A total of 55,381 patients who underwent a total thyroidectomy were identified comprising of 14,055 inpatient and 41,326 outpatient procedures. A cohort of 13,496 patients who underwent outpatient surgery were propensity matched for covariates with corresponding number of patients who underwent inpatient thyroidectomies. In the propensity matched cohort, the occurrence of any 30-day after surgery complications were greater in the inpatient group, 424 out of 13,496 (3.1%) compared to the outpatient group, 150 out of 13,496 (1.1%),* P* < 0.001. Moreover, death rates were greater in the inpatient group, 22 out 13,496 (0.16%) compared to the outpatient group, 2 out of 13,496 (0.01%), *P* < 0.001. Similarly, hospital readmissions occurred with greater frequency in the inpatient group, 438 out of 13,496 (3.2%) compared to the outpatient group, 310 out of 13,496 (2.3%), *P* < 0.001.

**Conclusion:**

Thyroidectomy procedures performed in the outpatient setting had less rates of adverse events, including serious postoperative complications (e.g., surgical site infection, pneumonia, progressive renal insufficiency). In addition, patients who had thyroidectomy in the outpatient setting had less 30-day readmissions and mortality. Surgeons should recognize the benefits of outpatient thyroidectomy when selecting disposition of patients undergoing neck surgery.

## Background

As healthcare costs continue to rise, there has been increased demand in the utilization of outpatient surgeries for increasingly invasive procedures (Hollenbeck et al. 2015; Molins et al. 2008). The advance of surgical and anesthesia techniques in combination with increasing pressure from insurance carriers have resulted in a substantial increase in thyroidectomies performed in the outpatient setting (Grubey et al. 2018; Philippe et al. 2019). Nonetheless, the safety of outpatient thyroidectomy is not well established and some surgeons still prefer to have patients admitted to a hospital after surgery (Balentine and Sippel 2016).

Some surgeons fear that life-threatening complications of missed hematomas or severe hypocalcemia warrant hospital admission increasing length of stay (LOS) to mitigate any post-operative complications. A recent multistate cross-sectional review was completed by Orosco et al*.* which reported the 30-day revisit rate at 7.2%, with relatively high frequencies occurring over a week post-operatively (Orosco et al. 2015). One can argue that the traditional average length of stay of 72 h would ultimately miss most of these postoperative complications.

The primary purpose of the present study was to compare 30-day complication rates for outpatient compared to inpatient thyroidectomies while controlling for differences in patient comorbidities and surgery characteristics. We hypothesized that there would be no difference in complication rates between outpatient and inpatient thyroidectomy procedures. In addition, we also sought to determine if there was a difference if postoperative readmissions and mortality between the study groups.

## Methods

This study was performed under an exempt status granted by the Institutional Review Board of Rhode Island Hospital (IRB#1532652). The IRB determined that the study qualified for exemption under 45 CFR 46.101(b). The exemption was granted because the study involved a retrospective review of existing data recorded in such a manner that subjects cannot be identified, directly or through identifiers linked to the subjects. Clinical information of the subjects was obtained for the years between 2015 and 2018 from the American College of Surgeons (ACS) National Surgical Quality Improvement Program (NSQIP) database. The study is reported following the STOBE guidelines for reporting observational studies (Avery and Rotondi 2020).

The ACS-NSQIP database is a national prospective database that compiles voluntarily reported data from over 680 institutions in the United States. Over 1 million cases were submitted as part of the 2017 and 2018 update to the NSQIP database. Data is collected on over 300 variables that include preoperative risk factors, intraoperative variables and post-operative outcomes including complications up to 30 days after surgical procedures. Data collection has been previously described in detail (Raval and Pawlik 2018; Jiang et al. 2018). In brief, data are collected in 8-day cycles, with the first 40 procedures in the cycle included in the dataset. The most commonly performed procedures are capped at 5 within each cycle to increase procedure heterogeneity. Trained clinical nurses assigned at each site collect data for 30 days postoperatively using isolated telephone interviews and operative and clinical notes. Interrater reliability audits of selected participating sites help ensure the collected data are of the highest quality possible. The combined results of inter-rater reliability audits completed to date revealed an overall inter-rater disagreement rate of approximately 1.8% for all assessed program variables (Raval and Pawlik 2018; Jiang et al. 2018).

De-identified patient information is freely available to all institutional members who comply with the ACS NSQIP Data Use Agreement. The Data Use Agreement implements the protections afforded by the Health Insurance Portability and Accountability Act of 1996 and the ACS NSQIP Hospital Participation Agreement. The ACS NSQIP and the hospitals participating in this program are the sources of the data used in this study; however, these entities have not verified and are not responsible for the statistical validity of the data analysis or the conclusions derived by the authors.

The 2015 through 2018 NSQIP Participant Use Data Files were queried to extract all patients scheduled to undergo total thyroidectomy. Patients who underwent primary, total thyroidectomy were identified using the Current Procedural Terminology (CPT) codes 60220, 60225, 60240 and 60252. We excluded CPT codes 60210 (partial total lobectomy) and 60212 (partial total lobectomy with contralateral subtotal lobectomy), and 60260 (completion thyroidectomy) because we wanted to only include patients undergoing primary, total thyroidectomy for comparison. We also excluded CPT codes 60254, 60270, and 60271 as these represent more extensive surgery (e.g., neck dissection) and will very often require patient admission.

### Outcomes variables and analysis

Preoperative demographic variables such as age, sex, body mass index, American Society of Anesthesiologists (ASA) classification, smoking status, hypertension, diabetes, congestive heart failure, disseminated cancer, bleeding disorder and COPD were compared between the two cohorts. Intraoperative factors including surgical duration and relative value units (RVUs) were also compared between the cohorts. RVUs reflect the relative level of time, skill, training, and intensity required of a physician to provide a given service. RVUs therefore are a method for calculating the volume of work or effort expended by a physician in treating patients. The primary independent variable was if the surgical procedure was performed in an outpatient (length of stay  < 1 day) versus an inpatient (length of stay  ≥ 1 day) stetting.

The primary outcomes of interest included any 30-day adverse events, defined as any surgical or medical complication within 30 days of surgery (Khavanin et al. 2014, 2015). Other outcomes of interest included surgical complications (e.g., overall surgical site infection (SSI), [which includes superficial SSI, deep incisional SSI, organ space SSI], wound dehiscence), medical complications (e.g., pneumonia, unplanned intubation), VTE [deep venous thrombosis, pulmonary embolism], failure to wean, progressive renal insufficiency, acute renal failure, urinary tract infection, stroke or cerebrovascular accident, cardiac arrest, myocardial infarction, bleeding, sepsis/septic shock, death, readmission, and return to the operating room.

### Statistical analysis

Due to the observational (non-randomized) nature of this data, propensity score matching was used to minimize the effects of confounding when assessing differences in patient demographics and surgical characteristics between outpatient and inpatient thyroidectomies. The propensity score is the probability of treatment group conditional on observed baseline characteristics.

In this study, the probability for undergoing an inpatient thyroidectomy procedure (propensity score) was calculated for each patient based on age, sex, body mass index, diabetes, smoking status, dyspnea, chronic obstructive pulmonary disease, congestive heart failure, hypertension, disseminated cancer, steroid use, bleeding disorder, ASA classification, RVUs and surgical duration. Inpatient thyroidectomies were one-to-one matched without replacement to an outpatient thyroidectomy with the nearest propensity score, using a caliper of 0.10. If such a match was not available, the patient was eliminated.

Prior to matching, pre-operative demographics, comorbidities and surgical characteristics were compared using unpaired Student’s t test for continuous variables, and Chi-Square Test for binary variables. Pre-operative demographics were compared in the matched cohorts using paired t-tests for continuous variables, and McNemar’s Test for binary variables.

After propensity score matching, differences in outcome rates of the matched cohorts were assessed using McNemar’s test for matched data. Relative risks were calculated, as were risk differences. The rates of events between outpatient thyroidectomy and inpatient thyroidectomy were compared for outcomes at 30 days post procedure. To adjust for multiple endpoint testing, an adjusted p-value was calculated to correct for False Discovery Rate (FDR).

All statistical analyses were conducted with the use of SAS software version 9.4 (SAS Institute Inc., Cary, North Carolina).

## Results

**Fig. 1:**
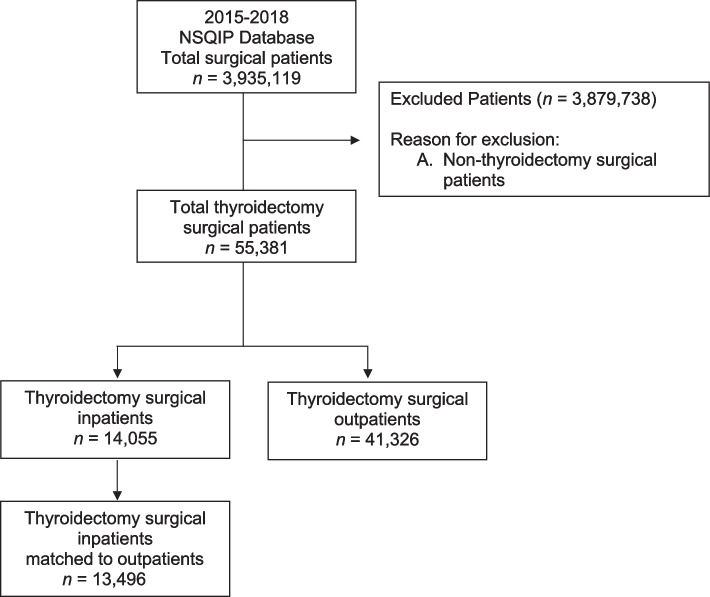
Study flow diagram. ACS-NSQIP indicates American College of Surgeons National Surgical Quality Improvement Program

A total of 55,381 patients undergoing thyroidectomy were included in the NSQIP database for 2015–2018. A total of 41,326 patients underwent outpatient thyroidectomy and 14,055 underwent inpatient thyroidectomies (Fig. 1). Of the 14,055 inpatient thyroidectomies,13,496 were propensity matched with 13,496 patients who underwent outpatient thyroidectomies. Patients in the original outpatient cohort group had greater BMIs, lower ASA classifications, and lower operative times (Table 1). Covariates were well balanced between the propensity matched cohorts, absolute standard mean difference  < 0.05 for all covariates (Table 2). Patient outcomes following thyroidectomy surgery prior to propensity matching is presented in Table 3.

**Table 1:** Baseline patient demographics and clinical characteristics^a^

	**All patients** **(*N* = 55,381)**	**Outpatient** **(*n* = 41,326)**	**Inpatient** **(*n* = 14,055)**	**Standard difference**	*P* Value*
Female, Sex, No. (%)	44557 (80.5)	33616 (81.3)	10941 (77.8)	0.087	< .001
Age, mean (SD), y	51.4 (14.8)	51.4 (14.6)	51.3 (15.2)	0.004	0.666
BMI, mean (SD)	30.7 (7.57)	30.8 (7.56)	30.3 (7.60)		< .001
Diabetes, No. (%)	7679 (13.9)	5554 (13.4)	2125 (15.1)	-0.048	< .001
Current Smoker, No. (%)	8030 (14.5)	5977 (14.5)	2053 (14.6)	-0.004	0.676
Dyspnea, No. (%)	3069 (5.5)	2203 (5.3)	866 (6.2)	-0.036	< .001
History of COPD, No. (%)	1397 (2.5)	1031 (2.5)	366 (2.6)	-0.007	0.475
Congestive Heart Failure, No. (%)	200 (0.4)	90 (0.2)	110 (0.8)	-0.080	< .001
Hypertension, No. (%)	21412 (38.7)	16030 (38.8)	5382 (38.1)	0.010	0.296
Disseminated cancer, No. (%)	382 (0.7)	232 (0.6)	150 (1.1)	-0.056	< .001
Steroid use, No. (%)	1483 (2.7)	1048 (2.5)	435 (3.1)	-0.034	< .001
Bleeding disorders, No. (%)	620 (1.1)	378 (0.9)	242 (1.7)	-0.071	< .001
ASA classification, No. (%)					
1 or 2	36,372/55266 (65.8)	27,647/41291 (66.9)	8725/13975 (62.4)	0.095	< .001
3, 4, or 5	18894/55266 (34.2)	13644/41291 (33.0)	5250/13975 (37.6)	-0.095	
RVUs, mean (SD)	15.03 (5.6)	14.6 (4.8)	16.18 (7.5)	-0.246	< .001
Operative time, mean (SD), No., min	113.4 (60.5), 55369	106.3 (52.2), 41318	134.1 (76.4), 14051	-0.424	< .001

^a^Includes patients in the American College of Surgeons National Surgical Quality Improvement Program database undergoing surgery for thyroidectomy of the neck from 2015 to 2018. Percentages have been rounded

^*^P-values were calculated from Chi-Square test for binary variables, and Students T-test for continuous variables

*Abbreviations*: *BMI* Body mass index, *COPD* Chronic obstructive pulmonary disease, *ASA* American Society of Anesthesiologists, *RVU* relative value unit

**Table 2:** Patient demographics and clinical characteristics after propensity matching^a^

	**All patients** **(*N* = 26,992)**	**Outpatient** **(*n* = 13,496)**	**Inpatient** **(*n* = 13,496)**	**Standard difference**	*P* Value*
Female, Sex, No. (%)	21121 (78.3)	10522 (77.9)	10599 (78.5)	-0.014	0.25
Age, Mean (SD), y	51 (14.9)	52 (14.8)	51 (15.1)	0.022	0.08
BMI, mean (SD)	30.4 (7.5)	30.4 (7.4)	30.3 (7.6)	0.012	0.32
Diabetes, No. (%)	4122 (15.3)	2086 (15.5)	2036 (15.1)	0.010	0.39
Current Smoker, No. (%)	3919 (14.5)	1950 (14.5)	1969 (14.6)	-0.004	0.74
Dyspnea, No. (%)	1628 (6.0)	817 (6.1)	811 (6.0)	0.002	0.88
History of COPD, No. (%)	683 (2.53)	338 (2.5)	345 (2.6)	-0.003	0.79
Congestive Heart Failure, No. (%)	161 (0.60)	81 (0.6)	80 (0.6)	0.001	0.94
Hypertension, No. (%)	10477 (38.8)	5308 (39.3)	5169 (38.3)	0.021	0.08
Disseminated cancer, No. (%)	244 (0.9)	119 (0.9)	125 (0.9)	-0.005	0.70
Steroid use, No. (%)	813 (3.0)	411 (3.1)	402 (3.0)	0.004	0.75
Bleeding disorders, No. (%)	431 (1.6)	220 (1.6)	211 (1.6)	0.005	0.66
ASA classification, No. (%)					
1 or 2	16901 (62.6)	8400 (62.2)	8501 (63.0)	-0.016	0.19
3, 4, or 5	10091 (37.4)	5096 (37.8)	4995 (37.0)		0.19
Sum of RVUs, mean (SD)	15.80 (6.2)	15.8 (6.3)	15.8 (6.0)	0.009	0.42
Operative time, mean (SD) min	128.3 (65.4)	128.1 (66.1)	128.5 (64.8)	-0.006	0.28

^a^Includes patients in the American College of Surgeons National Surgical Quality Improvement Program database undergoing surgery for thyroidectomy of the neck from 2015 to 2018. Percentages have been rounded

^*^P-values were calculated from McNemar’s test for binary variables and paired t test for continuous variables

*Abbreviations*: *BMI* Body mass index, *COPD* chronic obstructive pulmonary disease, *ASA* American Society of Anesthesiologists, *RVU* relative value unit

**Table 3:** Patient outcomes following thyroidectomy surgery prior to propensity matching^a^

	**Outpatient** **(*n* = 41,326)**	**Inpatient** **(*n* = 14,055)**	**Risk difference** **(95% CI)**	*P*-value*
Any 30-day complication	394 (0.1)	475 (3.4)	-2.43 (-2.74, -2.11)	< .001
Overall surgical complication	149 (0.4)	107 (0.8)	-0.40 (-0.56, -0.25)	< .001
SSI	144 (0.4)	95 (0.7)	-0.33 (-0.47, -0.18)	< .001
Superficial SSI	111 (0.3)	60 (0.4)	-0.16 (-0.28, -0.04)	0.005
Deep incisional SSI	21 (0.1)	20 (0.1)	-0.09 (-0.16, -0.03)	0.002
Organ/space SSI	12 (0.03)	15 (0.1)	-0.08 (-0.13, -0.02)	0.001
Wound dehiscence	5 (0.01)	14 (0.1)	-0.09 (-0.14, -0.03)	< .001
Overall medical complication	259 (0.6)	396 (2.8)	-2.19 (-2.47, -1.91)	< .001
Pneumonia	36 (0.1)	74 (0.5)	-0.44 (-0.56, -0.32)	< .001
Unplanned intubation	29 (0.1)	137 (1.0)	-0.90 (-1.07, -0.74)	< .001
VTE	34 (0.1)	34 (0.2)	-0.16 (-0.25, -0.07)	< .001
Deep vein thrombosis	17 (0.04)	19 (0.1)	-0.09 (-0.16, -0.03)	< .001
Pulmonary embolism	21 (0.1)	18 (0.1)	-0.08 (-0.14, -0.01)	0.005
Failure to wean	8 (0.02)	77 (0.6)	-0.53 (-0.65, -0.41)	< .001
Progressive renal insufficiency	2 (0.00)	14 (0.1)	-0.09 (-0.15, -0.04)	< .001
Acute renal failure	0 (0.00)	6 (0.04)	-0.04 (-0.08, -0.01)	< .001
Urinary tract infection	108 (0.3)	47 (0.3)	-0.07 (-0.18, 0.03)	0.166
Stroke/cerebrovascular accident	8 (0.02)	6 (0.04)	-0.02 (-0.06, 0.01)	0.135
Cardiac arrest	5 (0.01)	21 (0.2)	-0.14 (-0.20, -0.07)	< .001
Myocardial Infarction	11 (0.03)	16 (0.1)	-0.09 (-0.15, -0.03)	< .001
Bleeding	6 (0.01)	71 (0.5)	-0.49 (-0.61, -0.37)	< .001
Sepsis/Septic shock	29 (0.1)	40 (0.3)	-0.21 (-0.31, -0.12)	< .001
Sepsis	24 (0.1)	31 (0.2)	-0.16 (-0.24, -0.08)	< .001
Septic shock	5 (0.01)	10 (0.1)	-0.06 (-0.10, -0.01)	< .001
Death	12 (0.03)	23 (0.16)	-0.13 (-0.20, -0.07)	< .001
Readmission	807 (2.0)	463 (3.3)	-1.34 (-1.67, -1.02)	< .001
Return to the operating room	304 (0.7)	373 (2.7)	-1.92 (-2.20, -1.64)	< .001

^a^Includes patients in the American College of Surgeons National Surgical Quality Improvement Program database

undergoing surgery for thyroidectomy of the neck from 2015 to 2018. Percentages have been rounded

^*^P-values were calculated from McNemar’s test for matched data

*Abbreviations*: *SSI* surgical site infection, *VTE* venous thromboembolism

**Fig. 2:**
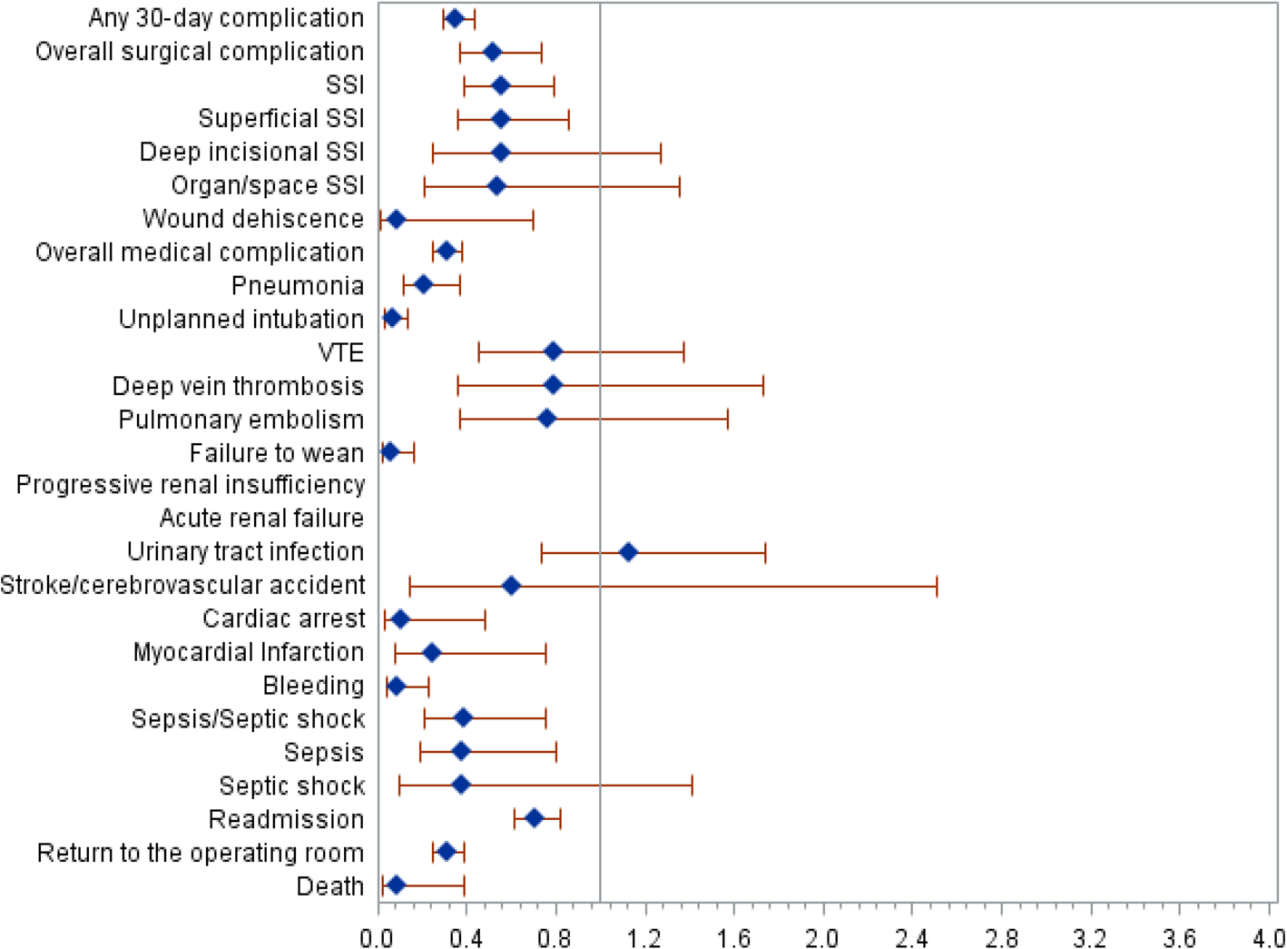
Forest Plots comparing the relative risk of postoperative complications between outpatient thyroidectomy and inpatient thyroidectomy procedures. Abbreviations: SSI = surgical site infection; VTE = venous thromboembolism; Diamonds represent the point estimate for relative risk; line represents 95% confidence intervals

In the matched cohorts, any 30-day complications were greater in the inpatient group, 424 out 13,496 (3.1%) compared to the outpatient group, 150 out of 13,496 (1.1%), *P* < 0.001. Many individual complications including overall surgical complication, surgical site infection, sepsis and myocardial infarction were greater in the inpatient compared to the outpatient thyroidectomies (Table 4). Relative risks for each individual complication comparing outpatient to inpatient thyroidectomies are presented on Fig. 2.

**Table 4:** Propensity matched patient outcomes following thyroidectomy surgery^a^

	**Patient group, No. (%)**		**Risk difference** **(95% CI)**	**Relative risk** **(95% CI)**	*P* Value*	**FDR** *P* Value**
	**Outpatient** **(*n* = 13,496)**	**Inpatient** **(*n* = 13,496)**				
Any 30-day complication	150 (1.1)	424 (3.1)	-2.03 (-2.37,-1.69)	0.35 (0.29,0.43)	< .001	< .0001
Overall surgical complication	49 (0.4)	95 (0.7)	-0.34 (-0.51,-0.17)	0.52 (0.37,0.73)	< .001	0.0003
SSI	48 (0.4)	86 (0.6)	-0.28 (-0.45,-0.11)	0.56 (0.39,0.79)	0.001	0.002
Superficial SSI	32 (0.2)	57 (0.4)	-0.19 (-0.32,-0.05)	0.56 (0.36,0.86)	0.008	0.012
Deep incisional SSI	9 (0.1)	16 (0.1)	-0.05 (-0.12,0.02)	0.56 (0.25,1.27)	0.16	0.208
Organ/space SSI	7 (0.1)	13 (0.1)	-0.04 (-0.11,0.02)	0.54 (0.21,1.35)	0.18	0.221
Wound dehiscence	1 (0.01)	11 (0.1)	-0.07 (-0.12,-0.02)	0.09 (0.01,0.70)	0.004	0.007
Overall medical complication	107 (0.8)	350 (2.6)	-1.80 (-2.11,-1.49)	0.31 (0.25,0.38)	< .001	< .0001
Pneumonia	14 (0.1)	67 (0.5)	-0.39 (-0.52,-0.26)	0.21 (0.12,0.37)	< .001	< .0001
Unplanned intubation	8 (0.1)	123 (0.9)	-0.85 (-1.02,-0.69)	0.07 (0.03,0.13)	< .001	< .0001
VTE	22 (0.2)	28 (0.2)	-0.04 (-0.15,0.06)	0.79 (0.45,1.37)	0.34	0.465
Deep vein thrombosis	11 (0.1)	14 (0.1)	-0.02 (-0.09,0.05)	0.79 (0.36,1.73)	0.55	0.570
Pulmonary embolism	13 (0.1)	17 (0.1)	-0.03 (-0.11,0.05)	0.76 (0.37,1.57)	0.47	0.518
Failure to wean	4 (0.03)	67 (0.5)	-0.47 (-0.59,-0.34)	0.06 (0.02,0.16)	< .001	< .0001
Progressive renal insufficiency	0 (0.00)	11 (0.1)	-0.08 (-0.13,-0.03)	–	< .001	0.002
Acute renal failure	0 (0.00)	4 (0.03)	-0.03 (-0.06,-0.00)	–	0.05	0.065
Urinary tract infection	44 (0.3)	39 (0.3)	0.04 (-0.10,0.17)	1.13 (0.73,1.74)	0.58	0.583
Stroke/cerebrovascular accident	3 (0.02)	5 (0.04)	-0.01 (-0.06,0.03)	0.60 (0.14,2.51)	0.48	0.518
Cardiac arrest	2 (0.01)	18 (0.1)	-0.12 (-0.18,-0.05)	0.11 (0.03,0.48)	< .001	0.0009
Myocardial Infarction	4 (0.03)	16 (0.1)	-0.09 (-0.15,-0.02)	0.25 (0.08,0.75)	0.007	0.012
Bleeding	5 (0.04)	55 (0.4)	-0.37 (-0.48,-0.26)	0.09 (0.04,0.23)	< .001	< .0001
Sepsis/Septic shock	13 (0.1)	33 (0.2)	-0.15 (-0.25,-0.05)	0.39 (0.21,0.75)	0.003	0.006
Sepsis	10 (0.1)	26 (0.2)	-0.12 (-0.21,-0.03)	0.38 (0.19,0.80)	0.008	0.012
Septic shock	3 (0.02)	8 (0.1)	-0.04 (-0.09,0.01)	0.38 (0.10,1.41)	0.13	0.178
Death	2 (0.01)	22 (0.2)	-0.15 (-0.22,-0.08)	0.09 (0.02,0.39)	< .001	0.0001
Readmission	310 (2.3)	438 (3.3)	-0.95 (-1.34,-0.56)	0.71 (0.61,0.82)	< .001	< .0001
Return to the operating room	110 (0.8)	351 (2.6)	-1.79 (-2.09,-1.48)	0.31 (0.25,0.39)	< .001	< .0001

^a^Includes patients in the American College of Surgeons National Surgical Quality Improvement Program database undergoing surgery for thyroidectomy of the neck from 2015 to 2018. Percentages have been rounded

^*^P-values were calculated from McNemar’s test for matched data

^**^False discovery rate was used to correct for multiple comparisons

*Abbreviations*: *SSI* Surgical site infection, *VTE* Venous thromboembolism, *FDR* False discovery rate

Moreover, death rates were greater in the inpatient group, 22 out 13,496 (0.16%) compared to the outpatient group, 2 out 13496 (0.01%), *P* < 0.001. Similarly, readmissions were greater in the inpatient group, 438 out of 13,496 (3.2%) compared to the outpatient group, 310 out of 13,496 (2.3%), *P* < 0.001.

## Discussion

The most important finding of the current investigation was the significant difference in postoperative outcomes between patients who had thyroidectomy surgery in the outpatient setting compared to the inpatient setting. Patients in the outpatient setting had lower postoperative complications including surgical site infection, sepsis and myocardial infarction. In addition, the rates of postoperative mortality and readmissions were lower in the outpatient cohort compared to the inpatient cohort. Taken together, our results suggest that the outpatient setting is a preferable pathway for patients having thyroidectomy.

Our results are clinically important given the current shift of practice towards the performance of thyroidectomy in the outpatient setting (Mallick et al. 2018; Black et al. 2017). Due to the current financial incentives and economic pressures to reduce costs in healthcare, it is expected that the number of outpatient thyroidectomy procedures will continue to growth over the following years (Sweet et al. 2020). Nonetheless, some clinicians still prefer to have their patients admitted to the hospital and stay more than twenty-four hours to monitor for potential complications including hypocalcemia and airway obstruction (Butler and Oltmann 2017; Jiang et al. 2019). Based on our results, the outpatient setting is not only adequate but a safer option for patients undergoing thyroidectomy.

Another important finding of our study was that patients undergoing outpatient thyroidectomy had lower rates of hospital readmissions than the patients who had inpatient procedures. It has been argued that keeping patients in the hospital can reduce readmissions due to hospital support (e.g., nursing care, intravenous medications) in the immediate postoperative period (Afflu et al. 2020; Schwartz et al. 2020). In the case of thyroidectomies, patients who had their procedure performed in an outpatient setting had less serious complications and this likely resulted in the less 30-day readmissions. It is well known that rates of nosocomial complications increase with greater hospitalization duration.

Prior studies comparing inpatient thyroidectomies to outpatient thyroidectomies have suggested that an outpatient setting has comparable safety to an inpatient setting. Mclaughlin et al*.* using data from the NSQIP database between 2005 and 2014 concluded that thyroidectomy performed as an outpatient was not found to be an independent risk factor for readmission or reoperation (McLaughlin et al. 2018). Orosco et al*.* examined a multistate database for outpatient procedures and concluded that ambulatory thyroidectomy demonstrates a good postoperative morbidity and mortality profile (Orosco et al. 2015). Our current results demonstrate that outpatient surgery is not only safe but likely the preferable pathway for patients undergoing thyroidectomy.

It was also interesting to note the selection process for the patients undergoing outpatient thyroidectomy compared to inpatient thyroidectomy. In the original cohort, patients in the outpatient setting had a greater rate of females and lower ASA classification. We used propensity score matching to adjust for the covariate imbalances in our analysis and obtained well-adjusted cohorts (e.g., standard mean difference  < 0.05 for all covariates). It is possible that clinical practitioners wanted to ensure better postoperative monitoring and more support to sicker patients (Aseni et al. 2019). Nevertheless, it is important to incorporate other factors (e.g., health literacy, family support) when selecting patients for ambulatory surgery to avoid readmissions (Jaffee et al. 2016; Wright et al. 2018).

Our study can only be interpreted within the context of its limitations. With a large, multi-institutional database such as the ACS-NSQIP, there are well published limitations including the possibility of clerical error, differences in interrater reliability across institutions and only a 30-day postoperative follow-up window. Due to limitations on the database, we cannot assess medication usage variations and hospital factors that could potentially alter the outcomes. For example, it is possible albeit unlikely, that patients in inpatient group received less antibiotic prophylaxis than the outpatient group and this explained the greater rates of surgical site infection. Compliance with updated national and hospital guidelines on surgical antibiotic prophylaxis may improve prescribing behavior. Lastly, the judgement for inpatient hospital admission is a complex medical decision based on the clinician’s judgement as well as the need for hospital care. Practice variations underlying the selection of inpatient versus outpatient surgery is not known which may be a contributing source of bias.

## Conclusion

In summary, after adjusting for covariate factors, thyroidectomy performed in the outpatient setting had less rates of adverse events, including serious postoperative complications (e.g., surgical site infection, pneumonia, progressive renal insufficiency). In addition, patients who had thyroidectomy in the outpatient setting had less 30-day readmissions and mortality than patients who had surgery in the inpatients setting. Surgeons should recognize the benefits of outpatient thyroidectomy when selecting disposition of patients undergoing thyroidectomy.

## Data Availability

The data that support the findings of this study are available from The American College of Surgeons National Surgical Quality Improvement Program but restrictions apply to the availability of these data, which were used under license for the current study, and so are not publicly available. Data are however available from the authors upon reasonable request and with permission of ACS NSQIP.

## Abbreviations

ACS: American College of Surgeons
ASA PS: American Society of Anesthesiologists physical status classification
CI: Confidence interval
CPT: Current procedural terminology
IRB: Institutional review board
LOS: Length of stay
NSQIP: National surgical quality improvement program database
OR: Odds ratio
RVU: Relative value unit
STROBE: Strengthening the reporting of observational studies in epidemiology
